# Access to HIV/AIDS care: a systematic review of socio-cultural determinants in low and high income countries

**DOI:** 10.1186/1472-6963-13-198

**Published:** 2013-05-28

**Authors:** Sara Gari, Camilo Doig-Acuña, Tino Smail, Jacob RS Malungo, Adriane Martin-Hilber, Sonja Merten

**Affiliations:** 1 Department of Epidemiology and Public Health, Swiss Tropical and Public Health Institute, Basel, Switzerland; 2 University of Basel, Basel, Switzerland; 3 Sophie Davis School of Biomedical Education, City University of New York, New York, USA; 4 International evaluation consultant, Particip GmBH, Freiburg, Germany; 5 Department of Population Studies, University of Zambia, Lusaka, Zambia

**Keywords:** Socio-cultural barriers, Access, Adherence, HIV/AIDS, Antiretroviral therapy, Survey study, Systematic review

## Abstract

**Background:**

The role of socio-cultural factors in influencing access to HIV/AIDS treatment, care and support is increasingly recognized by researchers, international donors and policy makers. Although many of them have been identified through qualitative studies, the evidence gathered by quantitative studies has not been systematically analysed. To fill this knowledge gap, we did a systematic review of quantitative studies comparing surveys done in high and low income countries to assess the extent to which socio-cultural determinants of access, identified through qualitative studies, have been addressed in epidemiological survey studies.

**Methods:**

Ten electronic databases were searched (Cinahl, EMBASE, ISI Web of Science, IBSS, JSTOR, MedLine, Psyinfo, Psyindex and Cochrane). Two independent reviewers selected eligible publications based on the inclusion/exclusion criteria. Meta-analysis was used to synthesize data comparing studies between low and high income countries.

**Results:**

Thirty-four studies were included in the final review, 21 (62%) done in high income countries and 13 (38%) in low income countries. In low income settings, epidemiological research on access to HIV/AIDS services focused on socio-economic and health system factors while in high income countries the focus was on medical and psychosocial factors. These differences depict the perceived different barriers in the two regions. Common factors between the two regions were also found to affect HIV testing, including stigma, high risk sexual behaviours such as multiple sexual partners and not using condoms, and alcohol abuse. On the other hand, having experienced previous illness or other health conditions and good family communication was associated with adherence to ART uptake. Due to insufficient consistent data, a meta-analysis was only possible on adherence to treatment.

**Conclusions:**

This review offers evidence of the current challenges for interdisciplinary work in epidemiology and public health. Quantitative studies did not systematically address in their surveys important factors identified in qualitative studies as playing a critical role on the access to HIV/AIDS services. The evidences suggest that the problem lies in the exclusion of the qualitative information during the questionnaire design. With the changing face of the epidemic, we need a new and improved research strategy that integrates the results of qualitative studies into quantitative surveys.

## Background

Socio-cultural diversity needs to be considered during the design of HIV/AIDS policies and programmes. Social attitudes and prejudices towards people living with HIV/AIDS, sexual taboos and gender inequality are some of the most important challenges for prevention and treatment of HIV/AIDS [1-6]. Despite an improved performance of health and community services, people living with HIV/AIDS continue to face persistent, deeply rooted, social and cultural barriers.

International donors, public health experts, programme planners and policy makers need to begin to recognize the need to take into account this socio-cultural diversity in program planning. Hence it is essential to gather the scientific evidence generated so far on this topic. Much of this evidence has been generated from qualitative studies. The most important and frequently reported socio-cultural barriers in both low income and high income countries include fear of disclosure, anticipation of stigma, limited social support, interpersonal violence and alcohol abuse [2-7]. To better understand the distribution, frequency, and potential impact that these factors may have on the population, quantitative epidemiological surveys should ideally incorporate similar questions. It is currently unclear to what extent socio-cultural determinants of access, identified by qualitative studies, are addressed in survey studies. To our knowledge there is no systematic review of epidemiological literature available to clarify this question. Therefore, this article seeks to answer three questions: What socio-cultural factors have been measured in epidemiologic studies to assess access to HIV/AIDS services? What are the differences between factors measured in low and high income countries? And what are the associations and effect sizes of these factors?

## Methods

A systematic search of quantitative studies addressing factors that influence access to HIV testing, uptake of antiretroviral therapy (ART) and adherence to antiretroviral (ARV) regimens was performed. In order to determine the differences between the factors studied in low and high income and test whether these differences were consistent with the findings of qualitative studies for each context we scrutinized studies from low and low-middle income countries and from upper-middle and high-income countries as defined by the World Bank Classification [8].

To facilitate the comparative analysis and the description of the results the countries belonging to these four income groups were reclassified into two broader categories: low and high income countries. The category low-income countries included low income countries and low-middle income countries and the high-income category included upper-middle and high-income countries.

### Search strategy

The search was restricted to studies with sample population over 18 years old and in English, French, German, Spanish, Portuguese and Italian. No other limitations were applied. The systematic search lasted one day with date 07^th^ October 2011. The search terms were: ‘HIV OR AIDS’, ‘voluntary counselling and testing’ ‘HAART OR antiretroviral*’, ‘compliance OR adherence’, ‘factors OR determinant* OR barriers’ and ‘motivat* OR facilitat*’. The search included Cinahl, EMBASE, CSA databases, IBSS, ISI Web of Science, JSTOR, MedLine, Psyinfo and Psyindex and the Cochrane Database of Systematic Reviews. Conference abstracts from the International AIDS Society conferences web site were also searched. We complemented the search by reviewing the bibliographies of key papers. The detailed search strategy is available upon request.

### Inclusion and exclusion criteria

To be eligible, articles needed to: report an original research study; measure one of these three outcomes: HIV testing, initiation of ART and adherence to antiretroviral therapy; study associations (of one of the three outcomes) with socio-cultural factors; target adult participants over 18 years old; apply a survey methodology for data collection; estimate risk effects as an outcome; and control for confounding in the analysis. Studies that reported socio-demographic or socio-economic factors but not any of the other categories of socio-cultural variables were excluded.

### Study selection and quality appraisal

The study selection followed a four-step process: title review; abstract review; full text review and quality appraisal. First, two of the authors independently reviewed all identified study titles. Duplicates and titles that did not meet the inclusion criteria were removed. The same authors then independently assessed the abstracts, and then the full papers of those abstracts that met the eligibility criteria. Finally, a quality appraisal was done on all full texts using consolidated criteria of the STROBE guidelines [9]. STROBE is a checklist of 22 items that must be addressed in the report of observational studies. This list is not really a tool to assess the quality of observational research but provides valuable guidance on the quality of reporting the studies.

In addition, a modified version of the Newcastle–Ottawa Scale (NOS) for observational studies (e.g. cross-sectional and cohort studies) was used to assess the methodological quality [10]. NOS is a tool to assess the quality of non randomized studies to be used in a systematic review. Each study is judged with a 'star system' on three points: the selection of the study groups, the comparability of the groups, and the ascertainment of the exposure or outcome. In our review, only studies in which five of nine items on the NOS were deemed satisfactory and in which appropriate statistical analysis (e.g. multivariate controlling for confounders) was conducted were considered to be of high methodological quality (maximum score of 9). At each stage of the quality assessment the reviewers discussed together until a consensus on which studies to include was reached. Finally, the reviewers manually searched the reference lists of the included articles for further key studies that could potentially be included in the analysis.

### Data extraction and classification

The following data was extracted and summarized in evidence tables: citation; year of publication; country; study design and sampling; characteristics of the study population; community versus facility based; sample; outcomes (HIV testing, uptake of ART, adherence and dropout); and factors that facilitated and/or hindered access to HIV care such as: socio-demographic; socioeconomic; medical; health system; knowledge and beliefs; risky health behaviours; psychosocial; stigma and discrimination; family and interpersonal violence; communication about HIV/AIDS; community prevalence. An overview of data extraction is provided in Tables 1 and 2.

**Table 1:** Characteristics of the studies

**Source**	**Year**	**Country**	**Design**	**Population**	**Setting**	**N**	**Outcome**
Aloisis	2002	Italy	Longitudinal	HIV + adults	Clinic	366	Adherence
Bardford	2005	Denmark	Cross sectional	HIV + adults	Clinic	887	Adherence
Boyer	2011	Cameroon	Cross sectional	HIV + adults	Clinic	2,381	Adherence
Carlucci	2008	Zambia	Cross sectional	HIV + adults	clinic	424	Adherence
Charurat	2010	Nigeria	Longitudinal	HIV + adults	Clinic	4,529	Adherence
Cunningham	1999	USA	Longitudinal	HIV + adults	Clinic	2,864	Initiation ART
de Castilho	2006	Brazil	Longitudinal	HIV + adults	Clinic	498	Adherence
Franke	2011	Peru	Longitudinal	HIV + adults	Clinic	132	Adherence
Gebo	2003	USA	Cross sectional	HIV + adults	Clinic	196	Adherence
Giday	2010	Ethiopia	Cross sectional	HIV + adults	Clinic	510	Adherence
Grierson	2011	Australia	Cross sectional	HIV + adults	Clinic	1,106	Adherence
Holmes	2007	USA	Longitudinal	HIV + adults	Clinic	116	Adherence
Karcher	2007	Kenya	Longitudinal	HIV + adults	Clinic	159	Initiation ART
Koku	2011	Ghana	Cross sectional	Female 15-49	Community	3,766	HIV testing
Kranzer	2010	South Africa	Longitudinal	HIV + adults	Clinic	1,154	Defaulting
Kranzer_b	2008	Malawi	Cross sectional	People 18-59	Community	2,047	HIV testing
Li	2010	Thailand	Cross sectional	HIV + adults	Clinic	386	Adherence
MacPhail	2009	South Africa	Cross sectional	People 15-24	Community	7,655	HIV testing
Martinez	2008	Uganda	Cross sectional	HIV + adults	Clinic	421	Initiation ART
Mugavero	2006	USA	Cross sectional	HIV + adults	Clinic	611	Adherence
Nam	2010	Vietnam	Cross sectional	HIV + adults	Clinic	353	Initiation ART
Okonsy	2011	USA	Cross sectional	HIV + adults	Clinic	558	Adherence
Peltzer	2011	South Africa	Cross sectional	HIV + adults	Clinic	735	Adherence
Pettifor	2010	South Africa	Cross sectional	People over 15 years old	Community	198	HIV testing
Pinheiro	2002	Brazil	Cross sectional	HIV + adults	Clinic	195	Adherence
Remien	2007	Brazil	Cross sectional	HIV + adults	Clinic	200	Adherence
Rintamaki	2006	USA	Cross sectional	HIV + adults	Clinic	204	Adherence
Sambisa	2010	Zimbabwe	Cross sectional	People over 15 years old	Community	12,254	HIV testing
Sarna	2008	India	Cross sectional	HIV + adults	Clinic	310	Adherence
Sayles	2006	USA	Longitudinal	HIV + adults	Clinic	1,910	Adherence
Sayles	2009	USA	Cross sectional	HIV + adults	Clinic	202	Adherence
Van Servellen	2005	USA	Cross sectional	HIV + adults	Clinic	85	Adherence
Wang	2007	China	Cross sectional	HIV + adults	Clinic	181	Adherence
Watt	2009	Tanzania	Cross sectional	HIV + adults	Clinic	340	Adherence

**Table 2:** Outline of the factors identified per study

**Country group**	**Source**	**Clinical**	**Disclosure**	**Location**	**Violence**	**Risk behavior**	**Health system**	**Health Beliefs**	**Psychosocial**	**Social support**	**SES**	**Stigma**
**Low and low middle income countries**	Boyer	x	.	.	.	x	x	.	.	x	x	x
	Carlucci	x	.	.	.	.	x	.	.	.	.	x
	Charurat	x	x	.	.	.	x	.	.	.	x	.
	Franke	x	.	.	.	x	.	.	x	x	x	x
	Giday	x	.	.	.	x	.	.	.	x	x	.
	Karcher	x	.	.	.	.	.	.	.	.	.	.
	Koku	.	.	.	.	x	.	x	x	.	x	x
	Martinez	x	.	.	.	x	.	.	x	.	.	.
	Nam	x	x	.	.	x	x	.	.	x	x	.
	Sarna	x	.	.	.	.	x	.	x	.	x	.
	Watt	.	.	.	.	.	x	.	x	.	.	.
	Kranzer_b	.	.	x	.	.	.	.	.	.	x	.
	Sambisa	.	.	.	.	x	.	x	.	.	x	x
**High and upper middle income countries**	Aloisis	x	.	.	.	x	.	.	.	.	.	.
	Bardford	x	.	.	.	x	x	.	x	x	.	.
	Cunningham	.	.	.	.	.	.	.	x	.	x	.
	De Castilho	x	.	.	.	x	.	.	x	.	.	.
	Gebo	.	.	.	.	x	.	x	.	x	x	.
	Grierson	x	.	.	.	x	x	x	x	.	.	x
	Holmes	.	.	.	.	x	.	.	.	.	x	.
	Kranzer	x	.	.	.	.	.	.	.	.	.	.
	Li	x	x	.	.	.	x	.	x	x	x	x
	Mugavero	.	.	.	.	x	.	.	x	.	x	.
	Okonsy	x	.	.	.	.	.	.	.	x	.	.
	Peltzer	x	.	.	.	x	.	.	x	x	x	x
	Pettifor	x	x	.	.	.	x	x	.	.	.	x
	Pinheiro	x	.	.	.	.	.	.	.	.	x	.
	Remien	x	.	.	.	.	.	.	x	.	.	.
	Rintamaki	x	.	.	.	.	.	.	.	.	.	x
	Sayles _a	.	.	.	.	.	.	.	x	.	.	x
	Sayles_b	x	.	.	x	x	.	.	x	.	x	.
	Vanservellen	x	.	.	.	.	x	x	x	x	.	.
	Wang	x	.	.	.	.	x	.	.	.	.	.
	Mc Phail	x	x	.	.	x	.	x	.	.	.	x

The data was extracted and reviewed in duplicate from identified studies. Common indicators were grouped into bigger categories (factors) in duplicate by independent reviewers. Disagreements in the categorization of the factors were discussed until consensus was reached. Countries of the study were classified as high or low income countries as defined by the World Bank [8]. Odds ratio (OR) or similar estimates (e.g. relative risk, hazard ratio) and their respective confidence intervals for every unique risk estimate involving a specific indicator and the uptake of testing, initiating ART and adhering to ART were extracted when available.

### Statistical analysis

Descriptive statistics were used to examine patterns across countries with respect to: proportion (%) of factors studied in relation to access to HIV/AIDS care, estimated effect sizes (adjusted odd ratios) identified (protective *vs* risk) for each factor and the precision around the estimates (95% confidence intervals). Additionally meta-analysis was done for nine specific socio-cultural factors identified by the studies as statistically significantly associated with adherence to antiretroviral therapy. Despite the expected heterogeneity within the review (great variability of the measures used to study socio-cultural factors) we assessed the comparability of the results from individual studies using the I^2^ statistic for quantifying inconsistency. An overall I^2^ test-value greater than 60% was considered as indicative of a high level of heterogeneity for which statistical pooling was not appropriate. Further analyses included sensitivity analysis performed by removing the studies that contributed to the heterogeneity in the meta-analysis and subgroup analyses to compare high-income countries with low-income countries. A p-value of less than 0.05 was considered statistically significant. Analyses were performed in STATA 12.1.

## Results

### Study selection

**Figure 1:**
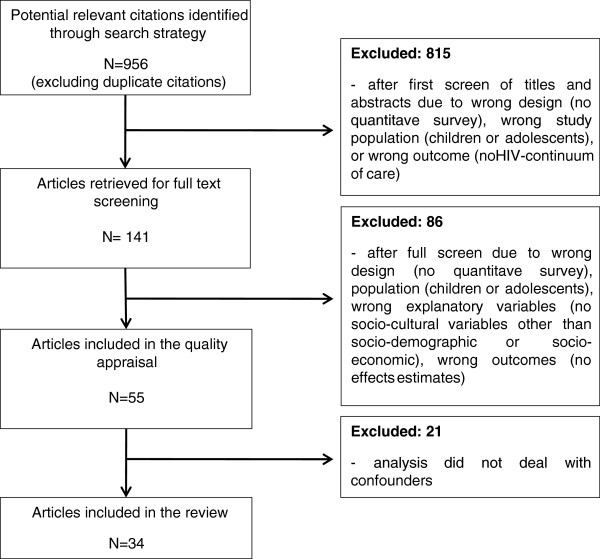
Flow chart.

The primary search strategy identified 1,671 potentially relevant citations. After searching for duplicates 715 citations were discarded. Initial title and abstract screening excluded 815 manuscripts based on the inclusion - exclusion criteria. The remaining 141 were then retrieved for full text review. A further 86 manuscripts were excluded as not meeting the inclusion criteria. The remaining 55 manuscripts were quality appraised and 21 were excluded as they did not deal with confounding in their analysis. Thirty-four articles were included in our analysis. Figure 1 displays the flow chart of the selection process.

### Study characteristics

All 34 included studies employed a quantitative methodology (surveys) and used structured questionnaires to determine potential factors. 13 studies (38%) were conducted in low income countries [11-23] and 21 (62%) in high income countries [24-44]. The studies conducted in low income countries included one from Cameroon [11], Zambia [12], Nigeria [13], Peru [14], Ethiopia [15], Kenya [16], Ghana [17], Uganda [18],^,^ Vietnam [19], India [20], Tanzania [21], Zimbabwe [22] and Malawi [23]. From high income countries, nine studies were from USA [26,28,30,33,34,39-42], four from South Africa [31,35,36,44], three from Brazil [27,37,38] and one each from Italy [24], Denmark [25], Australia [29], Thailand [32] and China [43].

A total of 29 studies (85%) were clinic based [11-16,18-21,24-35,37-40,42] and five (15%) were situated at community level [17,22,23,36,44]. Twenty-four studies (70%) focused on adherence to ART [11-15,20,21,24-30,32-39,41-43], five studies (15%) focused on uptake of voluntary and counselling testing (VCT) [17,22,23,36,44], four (11%) on ART initiation [16,18,19,26] and one (3%) on attrition [31]. Table 1 displays the characteristics of the studies.

### Factors measured by quantitative surveys to study access to HIV/AIDS-care in high- and low-income countries

12 factors were identified as measured by the studies to assess access to HIV care: (i) socio-demographic, (ii) socioeconomic, (iii) medical, (iv) health system, (v) knowledge and beliefs, (vi) risky health behaviours, (vii) psychosocial, (viii) stigma and discrimination, (ix) family (x) interpersonal violence, (xi) communication about HIV/AIDS and (xii) community prevalence. Table 2 shows an outline of the factors identified per study.

**Figure 2:**
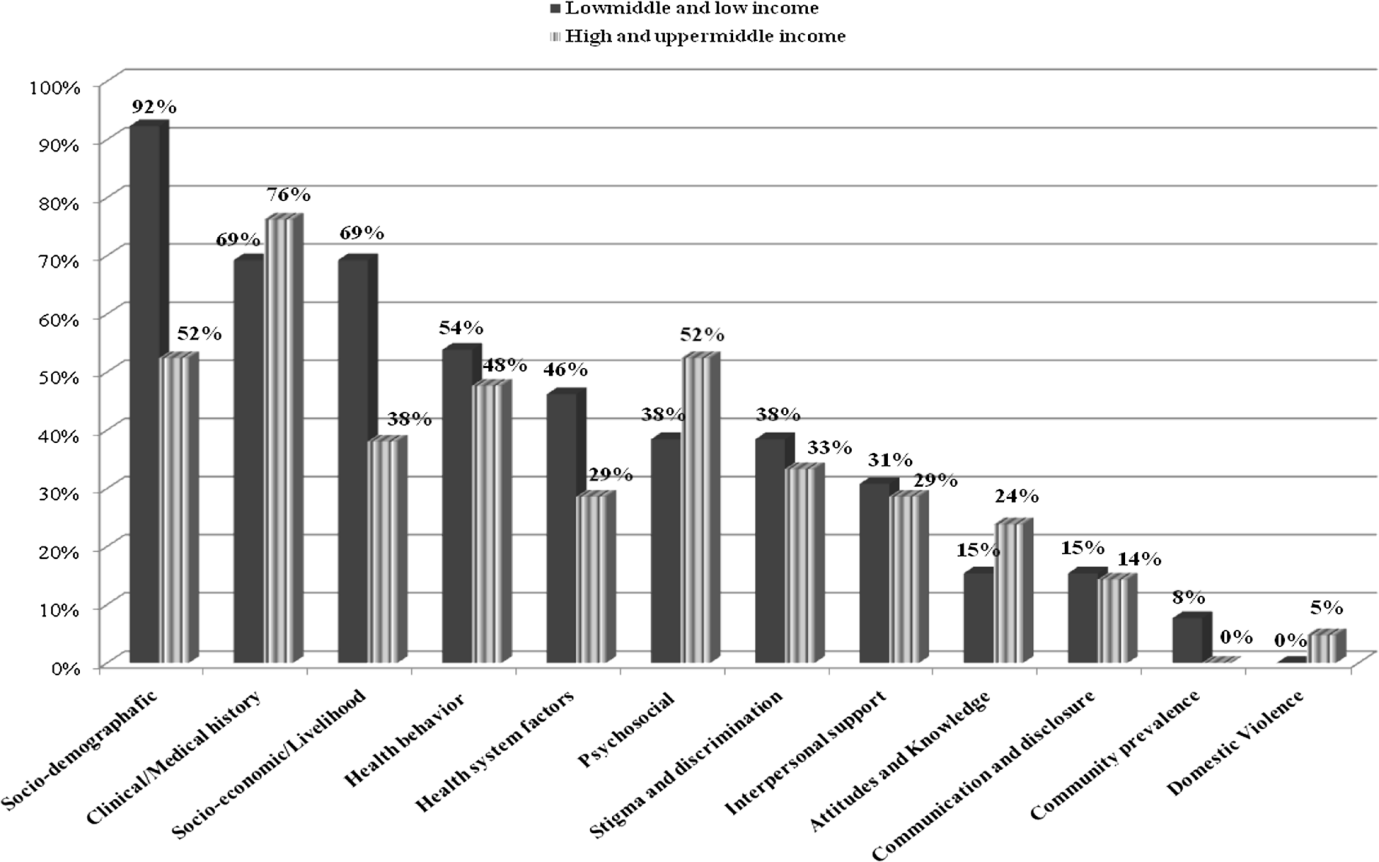
Proportion of factors (%) in studies carried out in HIC vs LIC.

Comparative analysis showed important divergences across countries. Surveys in low income countries tended to focus on the study of socio-demographic, socio-economic and health system factors in relation to access to HIV/AIDS services [24,25,27,29,31,32,34-40,42-44] while in high income countries the emphasis was on medical and psychosocial conditions [25-27,29,32,33,35,38,40-42]. Figure 2 graphically displays the distribution of factors by country. Socio-demographic, economic, clinical and sexual behavioural factors were considerably measured in all surveys conducted in low and high income countries while interpersonal relationships, communication and interpersonal violence factors remained highly understudied in both low and high economic countries. Table 3 displays the ranking of factors by the proportion of studies where they were included.

**Table 3:** Ranking of proportion (%) of factors studied in low and high income countries

**Low income countries**			**High income countries**		
**Rank**		**%**	**Rank**		**%**
1	**Socio-demographic factors**	**92%**	** 1**	**Clinical factors**	**76%**
2	**Clinical factors**	**69%**	** 2**	**Socio-demographic factors**	**52%**
3	**Socio-economic factors**	**69%**	** 3**	**Psychosocial factors**	**52%**
4	**Health behavior factors**	**54%**	** 4**	**Health behavior factors**	**48%**
5	**Health system factors**	**46%**	** 5**	**Socio-economic factors**	**38%**
6	Psychosocial factors	38%	6	Stigma and discrimination	33%
7	Stigma and discrimination	38%	7	Health system factors	29%
8	Social and family support	31%	8	Social and family support	29%
9	Knowledge and beliefs	15%	9	Knowledge and beliefs	24%
10	Communication and disclosure	15%	10	Communication and disclosure	14%
11	Community characteristics	8%	11	Domestic Violence	5%
12	Domestic Violence	0%	12	Community characteristics	0%

### Reported risk factors significantly associated with access to HIV/AIDS services across countries

Non-uptake of HIV testing in high income countries was associated with socio-demographic constructs such as being black [44], being between 25 to 34 years old, and living in a community with high HIV testing refusal rates [23]. Other barriers included high risk sexual behaviours [36] such as multiple sexual partners and not using condoms and enacted stigma [39]. In low income countries, the barriers associated to HIV testing included belonging to the age group from 25 to over 45 [17,22], having unprotected sex and having sex with a non-spousal or non-cohabiting partner [17] and anticipated stigma [17,22].

Non-uptake of ART in high income countries was only statistically significantly associated with having other competing subsistence needs [26]. In low income countries, the barriers to initiate ART included indirect costs of health care, not having a known HIV-positive family member, non-disclosure of HIV status and having additional pregnancies [11,19,20].

In high income countries, low adherence to ART was associated with being black [41,44], being between 25-34 years old [27,41] and having less than primary education [27]. Other barriers were clinical and treatment factors such as protease inhibitor ART regimens [34], frequent doses of ART [29,37], experience of side effects [34,38], feeling unhappy with the treatment [25], initiating the treatment with a CD4 count over 200 Cells/mL3 [31]. Alcohol and/or substance abuse [24,27-29,33] and anticipated and internalized stigma were associated with low levels of adherence [29,33,35,39].

In low income countries, age groups associated with low adherence to ART were 19-30 years and over 50 years [21]. The latter deviate from the risk age reported from high income countries where being over 50 was found to be protective. Other risk factors included having a main couple but not living together [11], being Muslim [13], being female [14], food insufficiency [14] and household financial problems [11,15]. Alcohol abuse and experienced discrimination were associated with low adherence also in low income countries [11]. With respect to clinical and treatment factors in low income countries initiation of ART with CD4 over 200 cells/ml3, being on ART less than 15 months [13,15] and having switched ART regimen [11] were associated with low adherence. Health system barriers included ARV stock outs, inadequate communication with health staff [11,13,21] and health care indirect costs [11,19,20]. One study reported an inverse association between free cost of ARV and adherence [20]. Concerning interpersonal and psychosocial factors, perceived lack of family support [11], not having disclosed to the family members [19] and feeling depressed [20] were negatively associated with adherence.

### Reported protective factors significantly associated with access to HIV/AIDS services across countries

In high income countries, protective factors for uptake of HIV testing were being female [36,44], having a history of previous illnesses [31,34,41,44], having disclosed and/or having conversations about HIV/AIDS with parents [44] and believe that most people do not want to get tested for HIV or do it only if they are sick [36,44]. In low income countries, being educated beyond primary school [17], single [22], affiliated to non Christian religions [17], living in a high prevalence community [22], knowing someone infected with HIV/AIDS [22], practicing safer sexual behaviours such as using condoms and being exposed to media [17,22] were positively associated with uptake of testing.

In low income countries, belonging to the age group 30 to 40 years of age and having greater than a primary school education facilitated initiation of ART [18]. Not drinking alcohol in the past year was also a protective factor [22]. No protective effects were reported from high income countries.

In high income countries, being older than 50 years of age [29] and on ART treatment for more than two years were positively associated with adherence to ART [35]. Previous illnesses or having other health conditions were positively associated with good adherence [31,34,41,44]. Self-perception of good health status [35,38] and no consumption of alcohol in the past year [30] were also protective. Good family communication [32,42], higher levels of treatment information [35] and believing in the benefits of ARVs [28] were facilitators of adherence. In low income countries, good social support and self-efficacy were positively associated with adherence as well as having disclosed to at least one family member about one’s positive HIV status [13,14]. Experiencing other health conditions was also associated with a protective effect on adherence [31,35,41,44].

No studies reported significant risk effects for defaulting.

### Combined effect sizes associated with adherence

The pool estimates of the 34 studies which included socio-cultural factors are shown for a) general socio-demographic factors, and b) specific socio-cultural factors. Due to an insufficient number of studies for other outcomes, only adherence could be included in the model.

The meta-analysis showed that being male was associated with optimal adherence in low income countries (OR= 0.16, 95%CI= 0.04-0.66) while the association with being female was not statistically significant. Conversely, in high income countries low adherence was associated with females (OR= 1.29, 95%CI: 1.06-1.58) while the association with male was not statistically significant. Being single (OR= 2.72, 95%CI= 1.58-4.69), and younger than 30 (OR= 1.04, 95%CI= 1.01-1.07) was significantly associated with lower adherence in low income countries. Being older than 50 years of age was associated with optimal adherence (OR= 0.80, 95%CI= 0.59-1.07) and was statistically significant in both settings; having no education was significantly associated with suboptimal adherence (OR= 1.76, 95%CI= 1.18-2.60) in both settings.

Socio-cultural factors associated with lower adherence included perceived lack of social support although sufficient data were available only in high income countries (OR= 1.04, 95%CI: 1.01-1.07). Perceived social stigma had an overall risk factor (OR= 2.17, 95%CI= 1.52-3.09) in both settings. High risk health behaviours such as alcohol abuse (OR= 1.75, 95%CI= 1.41-2.18) and abuse drugs (OR= 1.86, 95%CI= 1.48-2.33) were also significant in both settings, while low levels of self-efficacy were negatively associated with adherence in both settings. This effect was stronger in high income countries (OR=2.13 95%CI=1.03-4.41) than in low income countries (OR= 1.75 95%CI=1.91-1.31). Absence of symptoms of depression was positively associated with optimal adherence in both settings (OR= 0.89, 95%CI= 0.83-0.96). Tables 4 and 5 summarize the meta estimates of the socio-demographic and socio-cultural factors respectively.

**Table 4:** Meta-estimates: effect of socio-demographic factors on adherence to ART

**Socio-demographic**	**Pool ES**	**CI 95%**	p value*	**Pool studies**	**I**^**2**^
**Male**	0.77	0.48-1.24	0.28	7	61.3%
High income countries	0.94	0.57-1.55	0.81	4	54.6%
Low income countries	**0.16**	**0.04-0.66**	**0.01**	3	0.0%
**Female**	1.05	0.97-1.13	0.27	7	49.1%
High income countries	**1.29**	**1.06-1.58**	**0.01**	4	34.6%
Low income countries	0.99	0.91-1.08	0.85	3	0.0%
**Married**	1.10	0.85-1.42	0.46	2	0.0%
High income countries	1.10	0.84-1.45	0.49	1	-
Low income countries	1.10	0.56-2.16	0.78	1	-
**Single**	**2.49**	**1.51-4.12**	**0.00**	3	0.0%
High income countries	1.53	0.42-5.56	0.52	1	-
Low income countries	**2.72**	**1.58-4.69**	**0.00**	2	-
**Separated/divorced**	-	-	**-**	-	-
High income countries	-	-	**-**	-	-
Low income countries	1.07	0.42-2.73	0.89	2	0.0%
**Age less than 20**	1.14	0.96-1.37	0.14	2	60.1%
High income countries	0.47	0.96-1.37	0.13	1	-
Low income countries	**1.18**	**0.98-1.42**	**0.08**	1	-
**Age less than 30**	**1.04**	**1.01-1.07**	**0.01**	2	0.0%
High income countries	-	-	**-**	-	-
Low income countries	**1.04**	**1.01-1.07**	**0.01**	2	0.0%
**Age 20-30**	**1.38**	**1.21-1.58**	**0.00**	5	60.2%
High income countries	1.30	0.86-1.94	0.21	3	20.2%
Low income countries	**1.39**	**1.21-1.60**	**0.00**	2	74.0%
**Age 30-50**	1.08	0.97-1.19	0.16	6	0.0%
High income countries	1.06	0.77-1.46	0.72	4	0.2%
Low income countries	1.08	0.97-1.20	0.17	2	0.0%
**Age over 50 years**	0.80	0.59-1.08	0.14	5	59.4%
High income countries	**0.55**	**0.41-0.75**	**0.00**	4	0.0%
Low income countries	**6.68**	**3.15-14.15**	**0.00**	1	-
**No education**	**1.76**	**1.19-2.60**	**0.01**	3	0.0%
High income countries	1.69	0.82-3.48	0.16	1	-
Low income countries	**1.78**	**1.12-2.85**	**0.02**	2	0.0%
**Primary education**	0.98	0.85-1.13	0.80	4	9.3%
High income countries	0.99	0.86-1.15	0.91	3	0.0%
Low income countries	0.30	0.06-1.49	0.14	1	-
**Secondary education**	1.04	0.95-1.14	0.43	10	60.4%
High income countries	1.03	0.95-1.28	0.75	6	73.7%
Low income countries	1.04	0.94-1.15	0.47	4	19.8%
**Tertiary education**	0.85	0.58-1.24	0.39	3	60.9%
High income countries	0.69	0.45-1.07	0.10	2	44.8%
Low income countries	1.56	0.73-3.34	0.25	1	-

*Significance level p<0.05.

**Table 5:** Meta-estimates: effect of socio-cultural factors on adherence to ART

**Socio-cultural**	**Pool ES**	**CI 95%**	p value*	**Pool studies**	**I**^**2**^
**Low self-efficacy**	1.025	0.57-1.85	0.94	3	63.1%
High income countries	**2.13**	**1.03-4.41**	**0.01**	2	0.0%
Low income countries	**1.75**	**1.91-1.31**	**0.04**	1	-
**Lack of social support**					
High income countries	**1.04**	**1.01-1.08**	**0.02**	2	0.0%
Low income countries	-	-	**-**	-	-
**No depression**	**0.89**	**0.83-0.96**	**0.00**	2	0.0%
High income countries	0.92	0.82-1.04	0.17	1	-
Low income countries	**0.88**	**0.80-0.96**	**0.01**	1	-
**Stigma**	**2.17**	**1.52-3.09**	**0.00**	2	62.1%
High income countries	3.70	**1.92-7.42**	**0.00**	1	-
Low income countries	1.74	**1.14-2.65**	**0.01**	1	-
**Abuse of alcohol**	**1.75**	**1.41-2.18**	**0.00**	6	59.8%
High income countries	**1.43**	**1.09-1.86**	**0.01**	4	24.0%
Low income countries	**2.72**	**1.84-1.69**	**0.00**	2	32.7%
**Abuse of drugs**	**1.86**	**1.48-2.33**	**0.01**	9	1.1%
High income countries	**1.89**	**1.49-2.41**	**0.00**	6	23.2%
Low income countries	1.58	0.78-3.22	0.21	3	0.0%

*Significance level p<0.05.

## Discussion

This review revealed a trend in quantitative survey studies to explore the same kinds of factors in relation to access to HIV/AIDS services. Overall the most studied factors in all regions, including Africa, Asia, Latin America and some groups and communities in North America, were socio-economic, medical and health risk behaviour. In low-income countries the research focus was on socio-economic and health system factors while in high-income more attention was given to clinical and psychosocial factors such as depression, anxiety, self-efficacy and/or sexual identity. Socio-cultural factors including social and family support, interpersonal violence, and disclosure about HIV/AIDS received, in comparison, very little attention in both rich and poor countries.

These results should call the attention of survey researchers and systematic reviewers. The aforementioned socio-cultural factors have been widely published in qualitative studies [2-6] as critical factors that influence access to HIV/AIDS services, both in high and in low income countries. However most of the quantitative studies included in our review, from both high and low income countries, omitted them in their surveys or explored them very superficially. This is not justified as these factors are key issues for survey research. Our results suggest that the problem lied in the exclusion of qualitative information in the questionnaire design. Of the 34 studies included in this review, 27 [12-18,20,22-24,27,28,30-36,38-44] used validated measures from previous quantitative studies to derive their questionnaires and only seven studies, three, in low income countries [11,19,21] and four in high income countries [25,26,29,37], conducted an informative phase, using qualitative methods, to inform the questionnaire development.

The exclusion of qualitative information during the questionnaire design in the rest of the studies could have led to over-emphasis in the research of the same kind of easily measured variables.

This compromises the interpretation and generalization of the evidence and its application to inform health policies and programs. Indeed, this review showed that according to the quantitative evidence the factors studied to assess the barriers to access HIV/AIDS services inexplicably differ between richer and poorer countries contrary to the evidence from qualitative studies.

Additionally, due to an insufficient number of consistent studies for other outcomes, only adherence outcomes could be meta-analysed. The meta-analysis of the other outcomes proved untenable as the wide range of instruments and indicators used to assess socio-cultural variables such as social support, stigma, depression, and self-efficacy, introduced too much heterogeneity in the studies and impeded the pooling and synthesis of the results. Table 6 shows the variability of the instruments used to assess the same indicator. Risk factors of low-adherence, in both rich and poor countries, were stigma and discrimination, alcohol and drug abuse, depression and low self-efficacy. Social support was the only factor that showed a protective effect. Yet, it is unclear whether this effect occurred equally in rich and poor countries as enough data were available only from high income countries. The comparative approach between high and low income countries of this systematic review and meta-analysis has several advantages over pooling all countries included in the review. This comparative nature yielded valuable information about the differences and similarities of social and cultural processes that affect access to treatment in each context. The comparison also reveals a potential bias in the factors studied in the different regions that may be motivated by cultural stereotypes and has also facilitated the detection of trends and the identification of gaps in the surveys conducted which otherwise would have remained in the shadows. Thus this comparative approach has helped to produce a more detailed description of these gaps which can be beneficial for the preparation of future surveys in this field.

**Table 6:** Overview of measurement tools used to evaluate same socio-cultural constructs in different studies

**Source**	**Year**	**Country**	**Outcome**	**Measurement instrument**
**Family support**				
Boyer	2011	LIC	Adherence	Self-reported
Bardford	2005	HIC	Non adherence	Self-reported
Li	2010	HIC	Adherence	Adapted from FAD
**Social support**				
Vanservellen	2005	HIC	Adherence	MOS scale
Li	2010	HIC	Adherence	Adapted from MOS Scale
Franke	2011	LIC	Adherence	Duke-UNC Functional Social Support Questionnaire
Peltzer	2010	HIC	Adherence	Adapted Social Support Questionnaire
Okonsy	2011	HIC	Adherence	Rates social support 1 to 10
Giday	2010	LIC	Adherence	Own questions
**Self-efficacy**				
Franke	2011	LIC	Adherence	ACTG
Remien	2007	HIC	Adherence	ACTG
Vanservellen	2005	HIC	Adherence	ACTG
Watt	2009	LIC	Adherence	10 item scale adapted
**Depression**				
Martinez	2008	LIC	Initiation ART	Hopkins Symptoms Checklist
Franke	2011	LIC	Adherence	Hopkins Symptoms Checklist
Peltzer	2010	HIC	Adherence	CES-D
Vanservellen	2005	HIC	Adherence	CES-D
Mugavero	2006	HIC	Adherence	BSI
Sarna	2008	LIC	Adherence	BDI
Bardford	2005	HIC	Non adherence	ACTG
Li	2010	HIC	Adherence	Thai Department of Mental Health
				
**Source**	Year	Country	Outcome	Measurement instrument
**Patient-provider relationship**				
**Bardford**	2005	HIC	Non adherence	Self-reported
**Vanservellen**	2005	HIC	Adherence	Satisfaction survey
**Watt**	2009	LIC	Adherence	9 item scale adapted from Panpanich 2004
**Quality of life**				
**Franke**	2011	LIC	Adherence	Medical Outcomes Study HIV Health Survey
**Peltzer**	2010	HIC	Adherence	WHOQOL-HIVBREF
**Stigma**				
**Rintamaki**	2006	HIC	Adherence	3 items from PMAQ
**Franke**	2011	LIC	Adherence	Berger Scale
**Carlucci**	2011	LIC	Adherence	Own scale
**Pettifor**	2004	HIC	HIV testing	Genberg scale
**Li**	2010	HIC	Adherence	Adapted from Herek and Capitanio
**Sayles _a**	2009	HIC	Adherence	Own scale
**Koku**	2011	LIC	HIV testing	2003-GDHS
**Dicrimination**				
**Boyer**	2011	LIC	Adherence	Self reported
**Grierson**	2011	HIC	Adherence	Self reported
**Peltzer**	2010	HIC	Adherence	Own 7 items scale
**Pettifor**	2004	HIC	HIV testing	Genberg scale

There are several limitations to our study. Publication bias may be limiting our systematic review of quantitative studies although we have used Preferred Reporting Items for Systematic Reviews and Meta-Analyses (PRISMA) guidelines [45] to examine reporting and other biases in a systematic way. See Additional file 1 for further details. Another limitation is the difference between the timing of the preparation of the surveys and the one of the publications of qualitative studies. We would not expect a survey published in the early 90s to be aware of the problems identified in qualitative studies published later. But our analysis indicates that in general the quantitative studies have not systematically addressed important issues identified in qualitative studies that were published at least two years before the implementation of their surveys.

## Conclusions

This review has highlighted a number of issues requiring further research and demonstrated the need to improve the research strategy in epidemiological survey studies. Improvement of this strategy requires better integration of the findings of qualitative studies in quantitative surveys and more consistency between survey studies. This review also offered evidence of the lack of consistency in the measurement of socio-cultural factors which hinders comparisons between studies.

We recommend that, prior to developing a questionnaire, literature reviews should be systematically carried out including qualitative studies. This would help to identify appropriate themes for the context avoiding the tendency to focus on the same topics. We further recommend using validated instruments giving priority to cultural adaptations over the development of new measures. We also call for a generalization of some variables without limiting the specificity of the various contexts. For example, it would be useful to report the effects of different types of stigma, as defined by subscales of validated tools, rather than global scores which cannot be disentangled and are less informative. Another example is the social support measure which could also be broken down by subtypes, material, emotional, etc.

Further quantitative research is needed on socio-cultural determinants of HIV testing, initiation of antiretroviral therapy and defaulting in both low- and high-income countries. More consistency between qualitative and quantitative research and between quantitative measures of socio-cultural factors will help to increase the quality of the data collected, to enhance comparability which is a prerequisite for meta-analyses, to avoid duplication and in general to produce better scientific evidence to inform managers and policy-makers working on HIV/AIDS.

## Competing interests

The authors declare that they have no competing interests.

## Authors’ contributions

Conceived and designed the study: SG, SM, JM, AMH. Performed the systematic searches and abstracted data: SG, CD, TS. Analyzed the data: SG, TS. Wrote the paper: SG, SM, AMH, JM, TS, CD. All authors have read and approved the final manuscript.

## Pre-publication history

The pre-publication history for this paper can be accessed here:

http://www.biomedcentral.com/1472-6963/13/198/prepub

## Supplementary Material

Additional file 1 PRISMA 2009 Checklist. Click here for file
