# Supplementary material for: Access to HIV/AIDS care: a systematic review of socio-cultural determinants in low and high income countries
Source: BMC Health Serv Res. 2013 May 28;13:198. doi: 10.1186/1472-6963-13-198 (PMC3679910; doi:10.1186/1472-6963-13-198)
Supplement: Additional file 1 — PRISMA 2009 Checklist. [file 1472-6963-13-198-S1.docx]

Additional file 1 - Prisma 2009 Checklist

| **PRISMA 2009 Checklist** |  |  | **Reported**  **in the manuscript** | **Comments** |
| --- | --- | --- | --- | --- |
| **TITLE** | **1** | Identify the report as a systematic review, meta-analysis, or both. | Yes | Access to HIV/AIDS care: a systematic review of socio-cultural determinants in low and high income countries. |
| **ABSTRACT** |  |  | Yes |  |
| Structured summary | **2** | Provide a structured summary including, as applicable: background; objectives; data sources; study eligibility criteria, participants, and interventions; study appraisal and synthesis methods; results; limitations; conclusions and implications of key findings; systematic review registration number. | Yes | **On page 2- line 24-53.** |
| **INTRODUCTION** |  |  | Yes |  |
| Rationale | **3** | Describe the rationale for the review in the context of what is already known. | Yes | **On page 3- line 56-70.** |
| Objectives | **4** | Provide an explicit statement of questions being addressed with reference to participants, interventions, comparisons, outcomes, and study design (PICOS). | Yes | **On page 3- line 70-76.** |
| **METHODS** |  |  | Yes |  |
| Protocol and registration | **5** | Indicate if a review protocol exists, if and where it can be accessed (e.g., Web address), and, if available, provide registration information including registration number. | N/A |  |
| Eligibility criteria | **6** | Specify study characteristics (e.g., PICOS, length of follow‐up) and report characteristics (e.g., years considered, language, publication status) used as criteria for eligibility, giving rationale. | Yes | **On page 3 and 4 - lines 79-89** we specify the study characteristics  **On page 4 - Lines 91-92** we present limitations considered (studies in adult population, languages, etc.). To clarify that no further limitations were applied to the search strategy this **sentence have been added in page 4-line 92**: No other limitations were applied.  **On page 4 - Lines 101-108** we present the "Inclusion and Exclusion Criteria". |
| Information sources | **7** | Describe all information sources (e.g., databases with dates of coverage, contact with study authors to identify additional studies) in the search and date last searched. | Yes | **On page 4- line 93** we specify the date of the search.  **On page 4- line 96-99** we provide a list of the databases searched. |
| Search | **8** | Present full electronic search strategy for at least one database, including any limits used, such that it could be repeated. | Yes | **On page 4- line 93-95** we provide a list with the search terms as they were used in the search code. **On** **page4- line 99-100** we specify that "The detailed search strategy is available upon request". With this sentence we want to encourage the readers to write to the corresponding author if they are interested in having the search strategy with more details. |
| Study selection | **9** | State the process for selecting studies (i.e., screening, eligibility, included in systematic review, and, if applicable, included in the meta‐analysis). | Yes | On page 4 and 5 - line 109-128: this is explained under the heading "Study Selection and quality appraisal". |
| Data collection process | **10** | Describe method of data extraction from reports (e.g., piloted forms, independently, in duplicate) and any processes for obtaining and confirming data from investigators. | Yes | On page 5 and 6 - line 138-144: this is explained under the heading "Data Extraction and classification". |
| Data items | **11** | List and define all variables for which data were sought (e.g., PICOS, funding sources) and any assumptions and simplifications made. | Yes | **On page 5-line 130-137** we have included " The following data were extracted and summarized in evidence tables: citation; year of publication; country; study design and sampling; characteristics of the study population; community versus facility based; sample; outcomes (HIV testing, uptake of ART, adherence and dropout); and factors that facilitated and/or hindered access to HIV care such as: socio-demographic; socioeconomic; medical; health system; knowledge and beliefs; risky health behaviours; psychosocial; stigma and discrimination; family and interpersonal violence; communication about HIV/AIDS; community prevalence. An overview of data extraction is provided in Table 1 and 2".  **On page 5-line 138:** We have deleted "*The indicators used to measure the factors that facilitated and/or hindered the access to the continuum of HIV/AIDS-care were extracted and review in duplicate…* " and substituted by "These data was extracted and reviewed in duplicate…". |
| Risk of bias in individual studies | **12** | Describe methods used for assessing risk of bias of individual studies (including specification of whether this was done at the study or outcome level), and how this information is to be used in any data synthesis. | Yes | **On page 4 and 5 - line 118-128:** under the heading "Study Selection and quality appraisal" we describe the NOS scale method which we used to assess the risk of bias during the selection process. |
| Summary measures | **13** | State the principal summary measures (e.g., risk ratio, difference in means). | Yes | On page 6 - line 146-159: we state the summary measures under the heading "Statistical analysis". On page 6 - line 147 it has been added the text (adjusted odd ratios) and in line 149 the text (95% confidence intervals) to make it clearer. |
| Synthesis of results | **14** | Describe the methods of handling data and combining results of studies, if done, including measures of consistency  (e.g., I2) for each meta-analysis. | Yes | **On page 6 -** **line 151-156** we have added the text Despite the expected heterogeneity within the review (great variability of the measures used to study socio-cultural factors) we assessed the comparability of the results from individual studies using the I^2^ statistic for quantifying inconsistency. An overall I^2^ test-value greater than 60% was considered as indicative of a high level of heterogeneity for which statistical pooling was not appropriate. |
| Risk of bias across studies | **15** | Specify any assessment of risk of bias that may affect the cumulative evidence (e.g., publication bias, selective reporting within studies). | N/A | One of the main conclusions of this review is that the meta-analysis of the effects of socio-cultural factors have on access to HIV services is not feasible. Through the systematic review we identified a large diversity (heterogeneity) of methods used to measure the same socio-cultural constructs. Therefore, we chose not to use meta-analytic techniques with the exception of a few studies on adherence that examined factors more evenly. That is why it is in the results and discussion sections where we describe in more detail the risk of bias. **On page 9, line 263-264** under "Combined effect sizes associated with adherence".  **On page 11 lines 314-319.** Also in **Table 6** we show the different types of instruments used in the studies included in the review to measure the same type of factor, variability that makes impossible to poor their results for meta-analysis. |
| Additional analyses | **16** | Describe methods of additional analyses (e.g., sensitivity or subgroup analyses, meta-regression), if done, indicating which were pre‐specified. | Yes | **On page 6 - line 148-149** we have added the text Further analyses included sensitivity analysis performed by removing the studies that contributed to the heterogeneity in the meta-analysis and **in line 150** we specified the subgroup analysis. |
| **RESULTS** |  |  | Yes |  |
| Study selection | **17** | Give numbers of studies screened, assessed for eligibility, and included in the review, with reasons for exclusions at each stage, ideally with a flow diagram. | Yes | On page 6 - line 161-167 under "Study selection". Flow diagram represented in **Figure 1.** |
| Study characteristics | **18** | For each study, present characteristics for which data were extracted (e.g., study size, PICOS, follow-up period) and provide the citations. | Yes | On page 6 and 7 - line 169-182 under the heading "Study characteristics". Table 1 presents a comprehensive summary. |
| Risk of bias within studies | **19** | Present data on risk of bias of each study and, if available, any outcome level assessment (see item 12). | N/A | Only studies with low risk of bias were included in the review. This was a criteria of selection as explained **on page 4 and 5 - line 109-127** under the heading "Study Selection and quality appraisal". |
| Results of individual studies | **20** | For all outcomes considered (benefits or harms), present, for each study: (a) simple summary data for each intervention group (b) effect estimates and confidence intervals, ideally with a forest plot. | N/A | Our paper is primarily descriptive and mostly based on the results ofthe systematic review of studies (without meta-analysis). These results are extensively described in **pages 7 to 9-lines 183-260.** |
| Synthesis of results | **21** | Present results of each meta-analysis done, including confidence intervals and measures of consistency. | Yes | **On page 9-line 262-283** under "Combined effect sizes associated with adherence".  **On page 23-25-table 4 and 5**: a column showing the values of the I^2^ test has been added. |
| Risk of bias across studies | **22** | Present results of any assessment of risk of bias across studies (see Item 15). | Yes | Already explained in **item 15.** |
| Additional analysis | **23** | Give results of additional analyses, if done (e.g., sensitivity or subgroup analyses, meta-regression [see Item 16]). | N/A | Our paper is primarily descriptive and mostly based on the results ofthe systematic review of studies (without meta-analysis). These results are extensively described in **pages 7 to 9-lines 183-260.** |
| **DISCUSSION** |  |  |  |  |
| Summary of evidence | **24** | Summarize the main findings including the strength of evidence for each main outcome; consider their relevance to key groups (e.g., healthcare providers, users, and policy makers). | Yes | **On page 10 and 11- lines 286-333** under "Discussion". |
| Limitations | **25** | Discuss limitations at study and outcome level (e.g., risk of bias), and at review-level (e.g., incomplete retrieval of identified research, reporting bias). | Yes | **On page 12 –line 334-340** under "Discussion" (last paragraph). |
| Conclusions | **26** | Provide a general interpretation of the results in the context of other evidence, and implications for future research. | Yes | **On page 12 and 13-line 343-365** under "Conclusions". |
| **FUNDING** |  |  | Yes |  |
| Funding | **27** | Describe sources of funding for the systematic review and other support (e.g., supply of data); role of funders for the systematic review. | Yes | **On page 13-line 372-375** we have added a section on **Acknowledgements**: We are grateful to the Swiss National Science Foundation (SNSF) for the study funding. The funding organization played no role in the search, collection and interpretation of the data, and in the preparation, review, or approval of the manuscript. |
